# Supplementary material for: Information theoretical limits for quantum optimal control solutions: error scaling of noisy control channels
Source: Sci Rep. 2022 Dec 10;12:21405. doi: 10.1038/s41598-022-25770-6 (PMC9741659; doi:10.1038/s41598-022-25770-6)
Supplement: Supplementary file 1 — Supplementary Information. [file 41598_2022_25770_MOESM1_ESM.pdf]

Supplementary Information: Information Theoretical Limits for Quantum Optimal Control Solutions: Error Scaling of Noisy Control Channels

Matthias M. Müller[1,*], Stefano Gherardini[2,3], Tommaso Calarco[1,4], Simone Montangero[5], and Filippo Caruso[3]

[1]Forschungszentrum Jülich GmbH, Peter Grünberg Institute - Quantum Control (PGI-8), D-52425 Jülich Germany
[2]CNR-INO, Area Science Park, Basovizza, I-34149 Trieste, Italy
[3]Department of Physics & Astronomy & LENS, University of Florence, I-50019 Sesto Fiorentino, Italy
[4]Institute for Theoretical Physics, University of Cologne, D-50937 Cologne, Germany
[5]Department of Physics & Astronomy "G. Galilei", University of Padua, and with INFN Sezione di Padova, I-35131 Padua, Italy
[*]Corresponding author: ma.mueller@fz-juelich.de

ABSTRACT

In this supplementary Information we provide the derivation of the master equation under the evolution of a classical stochastic noise field (Eq. (10) of the main article.)

1 Derivation of the master equation (Eq. (10) of the main article)

Let us start the derivation from Eq. (8) in the main text, i.e.,

$$\dot{\rho}(t) = -i[H_s(t),\rho(t)] - i\left[\xi(t)H_n, \left(\rho(0) - i\int_0^t [H(t'),\rho(t')]dt'\right)\right].$$

After some straightforward calculations, one ends-up to the following relation:

$$\dot{\rho}(t) = -i[H_s(t),\rho(t)] - \xi(t)\left\{i\,[H_n,\rho(0)] + \int_0^t \Big[H_n,[H_s(t'),\rho(t')]\Big]dt'\right\} - \left[H_n,\left[H_n,\int_0^t \xi(t)\xi(t')\rho(t')dt'\right]\right].$$

In this way, by averaging over the noise realizations and using the assumption $\langle\xi(t)\rangle = 0$, one gets

$$\langle\dot{\rho}(t)\rangle = -i[H_s(t),\langle\rho(t)\rangle] - \left[H_n,\left[H_n,\int_0^t \langle\xi(t)\xi(t')\rho(t')\rangle dt'\right]\right],$$

where

$$\langle\xi_1(t)\rangle \equiv \int_{\xi_1} p_t(\xi_1)\xi_1\, d\xi_1$$
$$\langle\xi_2(t)\xi_3(t')\rangle \equiv \int_{\xi_2}\int_{\xi_3} p_{t,t'}(\xi_2,\xi_3)\xi_2\xi_3\, d\xi_2\, d\xi_3$$

with ξ_1, ξ_2, ξ_3 here representing generic stochastic processes. Accordingly, Eq. (10) in the main text is recovered under the further assumption to consider ξ and ρ as *uncorrelated* processes, so that the following approximation holds:

$$\int_0^t \langle\xi(t)\xi(t')\rho(t')\rangle dt' \approx \int_0^t \langle\xi(t)\xi(t')\rangle\langle\rho(t')\rangle dt' = \int_0^t R_\xi(t,t')\langle\rho(t')\rangle dt'.$$
